# Observational study of in-hospital mortality risk from bladder cancer: Five years of experience at a tertiary referral hospital in Indonesia

**DOI:** 10.1097/MD.0000000000039412

**Published:** 2024-08-23

**Authors:** Wahjoe Djatisoesanto, Yufi Aulia Azmi, Ida Bagus Gde Tirta Yoga Yatindra, Sony Wibisono Mudjanarko, Sri Umijati

**Affiliations:** a Department of Urology, Faculty of Medicine, Universitas Airlangga-Dr. Soetomo General Academic Hospital, Surabaya, Indonesia; b Faculty of Medicine, Universitas Airlangga, Surabaya, Indonesia; c Department of Health Sciences, University of Groningen, University Medical Center Groningen, Groningen, The Netherlands; d Department of Internal Medicine, Faculty of Medicine, Universitas Airlangga-Dr. Soetomo General Academic Hospital, Surabaya, Indonesia; e Department of Public Health, Faculty of Medicine, Universitas Airlangga-Dr. Soetomo General Academic Hospital, Surabaya, Indonesia.

**Keywords:** bladder cancer, diabetes, in-hospital mortality, oncology

## Abstract

Bladder cancer (BC) is a neoplasm arising from the bladder. It requires appropriate management and its prognosis depends on many factors. This study aimed to analyze the factors that influence outcomes in BC management. This was a retrospective study. Data were collected at one of Indonesia’s largest tertiary referral hospitals. All patients diagnosed with BC from January 2019 to December 2023 were included. The outcome measured was survival or death. Statistical analysis was conducted using SPSS version 26.0 software. The study included 219 patients with a median age of 57.97 years, of which 99 (45.2 %) patients died. In a bivariate analysis, sex, active smoking status, Karnofsky score, metastasis status, chronic kidney disease, type 2 diabetes mellitus, chemotherapy, radiotherapy, and alternative medicine were found to affect mortality status. Based on multivariate analysis, the route of admission (odds ratio [OR] 0.19), irregular visit (OR 6.21), metastasis (OR 3.58), radiotherapy (OR 21.12), and traditional medicine (OR 0.21) were independent factors of in-hospital mortality. The mortality rate for BC was considerably high. Irregular visits, metastasis, type 2 diabetes, and radiotherapy were independent risk factors for mortality.

## 1. Introduction

Any neoplasm that arises from the bladder is considered bladder cancer (BC), which is the most dominant form of urinary tract neoplasm.^[1,2]^ BC ranks as the 10th most frequent type of cancer globally. Each year, approximately 600,000 individuals are diagnosed with BC and over 200,000 people die of this condition worldwide.^[3,4]^ BC represents 3% of global cancer diagnoses and is highly prevalent in developed countries. Approximately 90% of BC diagnoses occur at age 55 years and over, and the prevalence of the condition is 4-fold higher in men than in women.^[4,5]^ The incidence rate in developing countries is doubled the developed countries.^[1,4]^

This cancer affects men and women. Certain risk factors, which include older age, being male, smoking, and exposure to toxins in the workplace and the environment, are associated with the development of BC. Because many cases are considered to have an environmental cause, BC is an appropriate target for preventive public health interventions. Cancer is a significant burden on the healthcare system because it results in considerable morbidity and death. Demographic trends, particularly population growth and aging, along with exposure to risk factors, such as smoking, affect the incidence of BC.^[4,6]^

BC is a common, significant, and costly disease. Screening studies suggest a survival benefit in asymptomatic populations with known risk factors.^[7,8]^ BC is a highly complex and expensive disease to both identify and treat. Diagnosis mostly relies on cystoscopy, which is an invasive and expensive procedure. The majority of BCs are recognized at an early stage, enabling effective treatment. However, more than a quarter of BCs are discovered at an advanced stage.^[3,9]^

Numerous factors determine the prognosis of BC.^[1,10]^ BC survival varies substantially between noninvasive and invasive cases based on stage. The number of noninvasive tumors is significantly high. Survival is linked to the stage, age, and histology.^[10–12]^ The accumulated chance of survival at 1, 3, 5, and 10 years in BC patients is 0.8989, 0.7132, 0.5752, and 0.2459, respectively. Survival rates differ tremendously according to age groups and the type of treatment provided.^[8,11,13]^ The stage and grade of cancer are crucial factors for selecting the ideal treatment for BC.^[2,14]^

Patients with BC might face in-hospital mortality. There are already 44 million cancer patients who have received their diagnosis >5 years ago. Each year, approximately 10 million individuals pass away from cancer. BC accounts for approximately 2.1% of all cancer-related fatalities and 3.0% of all new cancer diagnoses.^[4,15]^ Understanding the underlying factors can increase awareness and prevent poor prognosis. The aim of this 5-year observational study conducted at a single tertiary referral institution in Indonesia was to conduct an investigation into the risk factors related to in-hospital mortality in patients diagnosed with BC.

## 2. Methods

This study was conducted in compliance with the Helsinki Declaration, STROBE guidelines and gained approval from Dr Soetomo General Academic Hospital’s ethical review board (Approval number: 1527/LOE/301.4.2/XI/2023).^[16]^ As this was a retrospective study, written informed consent was not requested. This retrospective observational study has taken place at Dr Soetomo General Academic Hospital, the leading tertiary referral hospital in the east region of Indonesia. It included hospitalized BC patients throughout the 5-year interval between January 2019 and December 2023. Patients with BC who were adults were selected and those with incomplete data were excluded from the study.

## 3. Data collection

All data were sourced from the medical records. Patient’s age were divided with cutoff 60 years old, body mass index was divided based on WHO criteria into underweight (<18.5 kg/m^2^), normal (18.5–24 kg/m^2^), and overweight and obesity (>25 kg/m^2^), smoking category was divided into active smoker (smoke at least 1 cigarette per day for at least 6 month) and passive (nonsmoker but were exposed to daily cigarette fumes) based on self-report. Regular visit was defined as routine visit of at least once a month. Staging of BC was categorized based on American Joint Committee on Cancer 2017. Metastasis was defined as the spread of cancer cells from the primary tumor to other parts of the body. Comorbidities was defined as the presence of type 2 diabetes, hypertension, or chronic kidney disease (CKD). Type 2 diabetes was diagnosed if Hba1c > 6.5%, hypertension was diagnosed if the blood pressure is equal to or >140/90 mm Hg, taking antihypertension drugs, or had a history of hypertension. CKD was diagnosed if glomerular filtration rate was <60 mL/min per 1.73 m^2^, or the presence of kidney damage, or both, of at least 3 months Traditional medicine was defined as treatment other than that given by medical professional. Mortality was described as a report of death while the patient was admitted to the hospital.

## 4. Data analysis

Statistical analysis of this study was carried out with SPSS version 26.0 software (IBM Corp., Armonk, NY). The data normality was verified using the one-sample Kolmogorov–Smirnov test. The mean ± standard deviation is utilized for normally distributed data to portray the information. The median of interquartile range characterized skewed data, while the frequency (%) reported nominal data. The Fisher exact test, independent *t*-test, Mann–Whitney test, and chi-square test were chosen as suitable. Two-step logistic regression analyses were used to determine in-hospital mortality as the result of risk factor analysis. The initial stage involved the use of univariate logistic regression analysis to clinical characteristics and culture outcomes. The crude-odds ratio was acquired during this stage. The second phase included backward multivariate logistic regression analysis of all variables with *P*-values <.05 in the univariate study. An adjusted-odds ratio was calculated. In the multivariate logistic regression analysis, variables with a *P*-value <.05 were identified as independent risk factors for in-hospital death.

## 5. Results

Patients with BC (n = 219) admitted to the hospital between January 2019 and December 2023 were involved in this study. The cohort had a median age of 58 years, whereas 99 (45.2 %) patients died. The analysis revealed that sex, active smoking status, Karnofsky score, metastasis status, CKD, type 2 diabetes mellitus, chemotherapy, radiotherapy, and alternative medicine affected mortality status. Type 2 diabetes, chemotherapy, radiotherapy, and traditional medicine alone affected disease outcomes are illustrated in Table 1; however, the route of admission, regular visits, metastasis, staging disease, >2 comorbidities, type 2 diabetes, chemotherapy, radiotherapy, and traditional medicine were risk factors associated with BC mortality. Multivariate analysis of in-hospital mortality risk factors in patients with BC is represented in Table 2.

**Table 1:** Characteristics of patients with BC and independent risk factors for in-hospital mortality.

Variables	Total	Outcome		*P*-value	Crude odds ratio (95% CI)
		Survival	Non-survival		
Age in years, mean (SD)	57.97 (12,64)			.443	
Geriatric (>60 years old)				.988	0.996 (0.582–1.704)
Geriatric	95	52	43		
Adult	124	68	56		
Body mass index, median (IQR)	21.23 (3.32)			<.861	
Underweight	6	3	3		1.162 (0.796–1.388)
Normal	202	111	91		
Obesity	11	6	5		
Gender, n%				**.039**	**0.474 (0.231–0.972**)
Man	177 (80.8)	91	86		
Woman	42 (19.2)	29	13		
Route of admission				.537	0.843 (0.49–1.451)
Emergency room	130 (59.4)	69	61		
Policlinic	89 (41.6)	51	38		
Karnofsky Score, median (IQR)	90 (20)			**.001**	
Smoking category, n%				**.046**	**0.494 (0.245–0.995**)
Active	175 (79.9)	90	85		
Passive	44 (20.1)	30	14		
Regular visits, n%				.009	2.147 (1.208–3.816)
Yes	78 (35.6)	52	26		
No	141 (64.4)	68	73		
Staging disease				<.506	0.896 (0.705–1.274)
T2	73 (33.3)	36	37		
T3	72 (32.6)	42	30		
T4	74 (34.1)	42	30		
Metastasis				**.001**	**3.736 (2.127–6.564**)
Yes	124 (56.6)	122 (73.1)	2 (3.8)		
No	28 (12.8)	28 (16.8)	0		
Not yet staged	67 (30.6)	17 (10.2)	50 (96.2)		
Number of comorbidities				.815	1.113 (0.452–2.741)
>2	21 (9.6)	11	10		
>2	198 (90.4)	109	89		
Type 2 diabetes				**.001**	**4.597 (2.566–8.236**)
Yes	98 (44.7)	73	25		
No	121 (55.3)	47	74		
Hypertension				.707	0.881 (0.454–1.708)
Yes	77 (35.2)	23	21		
No	142 (64.8)	97	78		
Chronic kidney disease				**.001**	**0.285 (0.159–0.509**)
Yes	77 (35.2)	27	50		
No	142 (64.8)	93	49		
Chemotherapy				**.001**	**3.001 (1.692–5.324**)
Yes	88 (40.2)	62	26		
No	131 (59.8)	58	73		
Radiotherapy				**.001**	**10.292 (4.593–23.059**)
Yes	65 (29.7)	57	8		
No	154 (70.3)	63	91		
Radical cystectomy				.675	1.257 (0.431–3.661)
Yes	15 (6.8)	9	6		
No	204 (92.3)	111	93		
Traditional medicine				**.004**	**0.428 (0.238–0.77**)
Yes	67 (30.6)	27	40		
No	152 (69.4)	93	59		

CI = confident interval, IQR = interquartile range, SD = standard deviation.

**Table 2:** Multivariate analysis of in-hospital mortality risk factors in patients with BC.

Variables	*P*-value	Adjusted odds ratio (95% CI)
Unregular visits	**.001**	**6.205 (2.167–17.767**)
Metastasis	**.001**	**3.575 (1.670–7.651**)
Comorbidities > 2	.1	0.373 (0.116–1.207)
Type 2 diabetes	**.001**	**4.478 (2.070–9.687**)
Chemotherapy	**.007**	**0.179 (0.051–0.628**)
Radiotherapy	**.001**	**21.118 (5.453–81.788**)
Traditional medicine	**.001**	**0.212 (0.091–0.496**)

CI = confident interval.

## 6. Discussions

The highest mortality rates for BC, as stated by the database of the International Agency for Research on Cancer GLOBOCAN, are in Southern Europe (3.3 per 100,000) and Northern Africa (5.2 per 100,000), whereas the lowest rates were observed in Middle Africa (0.98 per 100,000) and Central America (0.84 per 100,000); however, GLOBOCAN figures have not specified risk factors, grading, or management.^[4,15,17]^ The current study found that the mortality rate for BC was 45.2%. The risk factors related to mortality included the route of admission, regular visits, metastasis, having >2 comorbidities, type 2 diabetes, chemotherapy, radiotherapy, and traditional medicine.

A systematic review and meta-analysis of 17 studies found that active smokers had an increased risk of death after radical cystectomy (hazard ratio [HR] 1.21, 95% confidence interval [CI] 1.08–1.36; *P* = .001), death from BC recurrence (HR 1.24, 95% CI 1.12–1.38; *P* < .001), and death from cancer (HR 1.24, 95% CI 1.13–1.36; *P* < .001).^[17]^ Smoking has also been related to a negative pathological impact on neoadjuvant chemotherapy, increasing the risk of metastatic development and mortality from BC.^[4,18,19]^; however, chemotherapy and smoking did not significantly affect the outcome.

The goal of screening for BC is to reduce mortality and increase survival rates through early detection of progression and metastases. Because most patients with muscle-invasive BC are diagnosed at an advanced stage, screening appears to be a promising approach. Early detection may increase survival rates and reduce the incidence of invasion and metastasis.^[17,20]^ Despite our finding that type 2 diabetes was a major risk factor for BC mortality, a recent meta-analysis indicated significant heterogeneity in the included studies (Q = 660.30, *P* < .001, I^2^ = 94.7%).

The overall relative risk (RR) with a 95% CI for the case–control and cohort studies was 1.45 (95% CI, 1.13–1.86) and 1.35 (95% CI, 1.35–1.62), respectively, when stratified by research type; however, cohort studies specifically focusing on diabetic individuals did not exhibit a significant correlation between diabetes and BC risk (RR = 1.25; 95% CI, 0.86–1.81).^[21]^ The observed heterogeneity was statistically significant for cohort studies (Q = 315.87, *P* < .001, I^2^ = 94.3%), case–control studies (Q = 22.13, *P* = .005, I^2^ = 63.8%), and patient studies (Q = 264.47, *P* < .001, I^2^ = 97.4%). The identification of BC in patients with CKD presents greater challenges compared with those having normal renal function using current urological and imaging techniques. In the present study, CKD did not significantly contribute to the risk of mortality. This may due to the fact that most CKD patient in this study coincidentally had routine control and in good condition, thus preventing their death. Number of comorbidities was not significant in this study because we did not evaluate the comorbidities specifically, therefore we could not stratify the severity of comorbidities that may affect the end result of this study

The association between the risk factors for metabolic syndrome and its parts and the incidence of BC and death exhibited variety; however, in cohort studies, it showed a strong relationship consistent with the meta-analysis of cohort studies. This meta-analysis revealed a statistically significant link between BC and metabolic syndrome 1.09 (95% CI, 1.02–1.17), and a significantly significant correlation existed between hypertension (RR, 1.07; 95% CI, 1.01–1.13) and diabetes (RR, 1.23; 95% CI, 1.16–1.31) with BC.^[22,23]^ Metabolic syndrome, which primarily includes obesity, hypertension, and diabetes mellitus, remains debatable as to whether it has a direct relationship with BC prognosis, although diabetes has an association that worsens prognosis.^[24]^ In a prospective study, scores on metabolic syndrome combined showed a moderately increased risk in men.^[25]^ There are 3 mechanisms by which diabetes increases the risk of BC: hyperglycemia, hyperinsulinemia, and urinary tract infections. Hyperglycemia causes dysregulation of energy balance, resulting in impaired immune system function and disturbed intracellular metabolism. Hyperinsulinemia causes an increase in insulin-like growth factors, thereby increasing the risk of enhanced cell proliferation and apoptosis inhibition.^[23]^ BC is the most frequent cancer of the urinary system, but its general incidence continues to be decreasing annually since 1992. In the present study, we identified 4 factors that were significantly associated with overall BC patient survival. Based on these factors, we developed and validated a nomogram that predicts overall individual BC patient survival.^[26]^ We also categorized patients into 2 prognostic groups with markedly different 6-month survival rates based on 3 possible prognostic factors: the number of locations affected by metastatic cancer, the initial American Joint Committee on Cancer stage, and the Karnofsky score. Our findings revealed that only 9% of patients with a total score of 6 to 8 survived for 6 months. Consequently, some individuals may be suitable candidates for brief, less intense radiation treatment. Several studies on palliative radiation treatment for solid tumor metastases, including BC, have demonstrated that even short-term irradiation regimens lasting a single day or a week can significantly alleviate symptoms.^[27]^ The use of Hypofractionated Palliative Radiation Therapy showed improvement in urinary frequency, nocturia, dysuria, and hematuria, which is promising for palliative therapy.^[28]^ Radical cystectomy was not found to be related with in-hospital mortality of BC in this study. This is probably due to only 6.8% of the patients undergo cystectomy, therefore more patients with cystectomy are needed to draw a conclusion.

## 7. Limitations

There are several limitations to this study. The retrospective design and single-center nature of this study constitute substantial limitations. Race, the type of chemotherapy provided, the amount of radiotherapy, type of the traditional medicine given and not all comorbidities were accounted for in this study, all of which may have contributed to bias in the results. However, we successfully conducted an initial epidemiological investigation on BC and the associated mortality risk factors in Indonesia, over an extended period with a large sample size. Our study lays the groundwork for further investigations in Asia, with a particular focus on Indonesia.

## 8. Conclusion

Our findings indicate that irregular visits, metastasis, type 2 diabetes, chemotherapy, radiotherapy, and traditional medicine were found as independent mortality risk factors in BC patients. Giving an additional attention towards these factors could improve the quality of life of the BC patient. A prospective study involving various institutes and larger samples are required to further validate these findings.

## Author contributions

**Conceptualization:** Wahjoe Djatisoesanto, Yufi Aulia Azmi.

**Formal analysis:** Yufi Aulia Azmi, Sri Umijati.

**Investigation:** Wahjoe Djatisoesanto, Yufi Aulia Azmi, Ida Bagus Gde Tirta Yoga Yatindra.

**Methodology:** Wahjoe Djatisoesanto, Sri Umijati.

**Resources:** Wahjoe Djatisoesanto, Yufi Aulia Azmi.

**Software:** Yufi Aulia Azmi, Sony Wibisono Mudjanarko.

**Validation:** Wahjoe Djatisoesanto.

**Writing – review & editing:** Wahjoe Djatisoesanto, Yufi Aulia Azmi.

**Writing – original draft:** Ida Bagus Gde Tirta Yoga Yatindra, Sony Wibisono Mudjanarko.
